# Nutrition Interventions in Rheumatoid Arthritis: The Potential Use of Plant-Based Diets. A Review

**DOI:** 10.3389/fnut.2019.00141

**Published:** 2019-09-10

**Authors:** Jihad Alwarith, Hana Kahleova, Emilie Rembert, Willy Yonas, Sara Dort, Manuel Calcagno, Nora Burgess, Lee Crosby, Neal D. Barnard

**Affiliations:** ^1^Physicians Committee for Responsible Medicine, Washington, DC, United States; ^2^Adjunct Faculty, George Washington University School of Medicine and Health Sciences, Washington, DC, United States

**Keywords:** autoimmune, diet, inflammation, plant-based, vegan, vegetarian, rheumatoid arthritis

## Abstract

Rheumatoid arthritis (RA), a chronic inflammatory autoimmune disease, affects roughly 1% of the world's population. RA pathogenesis remains unclear, but genetic factors account for 50–60% of the risk while the remainder might be linked to modifiable factors, such as infectious diseases, tobacco smoking, gut bacteria, and nutrition. Dietary triggers may play an inciting role in the autoimmune process, and a compromised intestinal barrier may allow food components or microorganisms to enter the blood stream, triggering inflammation. In addition, excessive body weight may affect pharmacotherapy response and the likelihood of disease remission, as well as the risk of disease mortality. Evidence suggests that changes in diet might play an important role in RA management and remission. Several studies have shown improvements in RA symptoms with diets excluding animal products. Studies have also shown that dietary fiber found in these plant-based foods can improve gut bacteria composition and increase bacterial diversity in RA patients, thus reducing their inflammation and joint pain. Although some of the trigger foods in RA patients are individualized, a vegan diet helps improve symptoms by eliminating many of these foods. This review examines the potential role of a plant-based diet in mediating RA symptoms. Further research is needed to test the effectiveness of plant-based diets on joint pain, inflammation, and quality of life in patients with RA.

## Introduction

Rheumatoid arthritis (RA), a chronic inflammatory autoimmune disease, affects roughly 1% of the world's population (1). Hands, wrists, and knees are most commonly bilaterally affected causing inflammation, pain, and eventually permanent joint damage (2). Genetic factors may account for a portion of risk (3–5), while the rest might be linked to environmental factors or a combination of genetic and environmental factors. Infectious diseases, tobacco smoking, and gut bacteria have all been considered to play a role in the development or progression of RA (6). Medications are a mainstay of treatment, but have unwanted side effects or are often expensive (7). Thus, changes in diet might be an easy and economical intervention in the management of RA.

Several studies have shown a correlation between modifiable risk factors and improvement of symptoms and outcomes in RA patients. Excessive body weight and diets that include animal products (e.g., dairy and red meat) tend to impair RA management efforts and exacerbate symptoms, presumably due to their pro-inflammatory effects (8). In contrast, diets rich in vegetables, fruits, and fiber are associated with lower BMI (9–11), have anti-inflammatory properties and help reduce pain and inflammation in these patients (12). Specifically, a 4-weeks low-fat vegan diet has been shown to significantly improve RA symptoms such as joint pain, stiffness, swelling and limitation in function (13). Likewise, a 1-year intervention tested the effects of a 7–10 day fast, followed by 3.5 months of a gluten-free vegan diet and gradual adoption of a vegetarian diet for the remainder of the study period. Significant improvements in several RA disease activity variables were observed after 1 month, including: number of tender joints, Ritchie's articular index, number of swollen joints, pain score, duration of morning stiffness, grip strength, erythrocyte sedimentation rate, C-reactive protein, white blood cell count, and a health assessment questionnaire score. These improvements were maintained after 1 year (14).

Several studies have reported lower risk of autoimmune diseases with a vegan diet. A 2013 study, using data from the Adventist Health Study-2 (AHS-2) cohort (*n* = 65,981), described a lower incidence and prevalence of hypothyroidism in people following vegan diets, compared to omnivorous, lacto-ovovegetarian, semi-vegetarian, and pesco-vegetarian diets even after controlling for BMI and demographic variables. The researchers speculated that the inflammatory properties of animal products could explain the lower risk in vegans (15).

Tonstad et al. also examined the correlation between dietary patterns and hyperthyroidism in the AHS-2 study population. Noting that the most common cause of hyperthyroidism is Graves' Disease, an autoimmune disorder, the researchers observed a 52% lower risk of hyperthyroidism with those consuming a vegan diet when compared to omnivores (16). Compared to non-vegetarians and lacto-ovovegetarians, vegans reported the lowest intake of saturated and *trans* fats, the highest intake of fiber, and displayed the lowest mean BMI (16), all of which could be relevant for the risk of hyperthyroidism. Potential down-regulation of insulin-like growth factor (IGF-1) (17) and higher consumption of polyphenols (18) in vegans are other possible protective mechanisms against hyperthyroidism.

Lauer et al. examined risk factors for multiple sclerosis, an autoimmune disease of the central nervous system, in male World War II veterans using the 1993 nationwide case-control study (*n* = 10,610) (19, 20). In the U.S, meat and dairy sales were significantly correlated with multiple sclerosis risk, while inverse associations were found with fruit and vegetable sales. Affluence was also positively associated with multiple sclerosis risk, corresponding with increased meat and dairy consumption with higher socioeconomic status.

These results suggest that a vegan diet, with a high intake of fruits and vegetables and the elimination of animal products, could protect against the development of autoimmune conditions. In contrast, diets high in animal products and low in fiber might increase the risk of developing these autoimmune conditions.

Intestinal gut health might play a role in the observed anti-inflammatory effects of dietary fiber. Studies have shown that dietary fiber can alter the composition of gut bacteria and increase the bacterial diversity, which is oftentimes lacking in RA patients, thus preventing intestinal damage (21).

Accumulating scientific evidence supports the health advantages of vegetarian diets (22). Vegetarian diets are characterized by reduced or eliminated consumption of animal products but may include dairy products and/or eggs, while vegan diets contain only plant foods. Both vegetarian and vegan diets typically emphasize vegetables, fruits, grains, legumes, and nuts. This paper summarizes the associations between diet and RA and makes a case for the potential benefits of a vegan diet in RA management.

## Pathogenesis of Rheumatoid Arthritis

**Figure 1:**
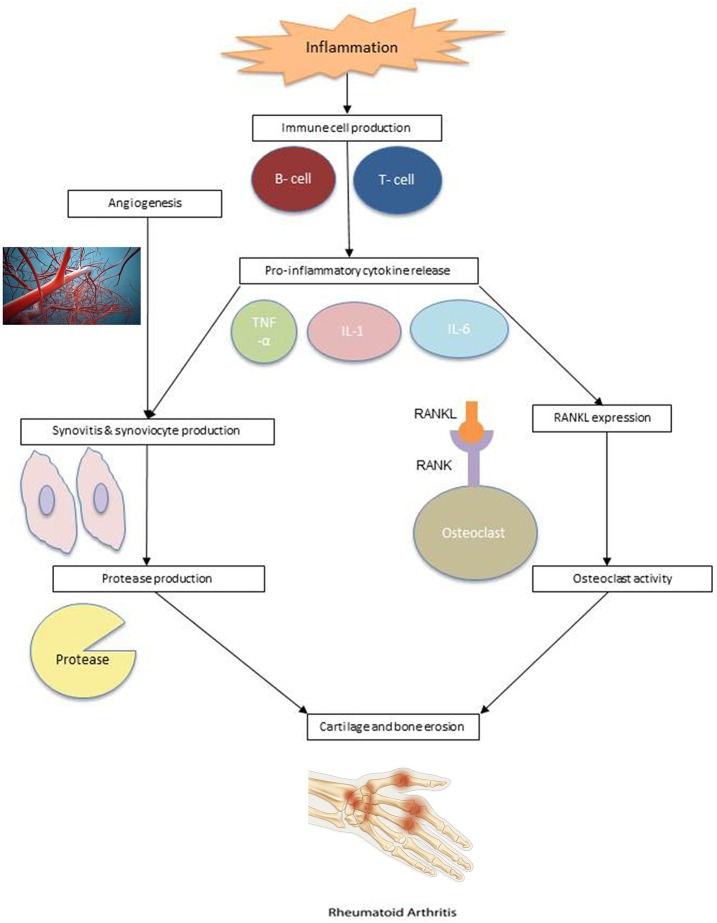
RA pathogenesis. Angiogenesis: Reproduced from Sitox / E+ via Getty Images (https://www.gettyimages.com/detail/photo/vascular-system-veins-royalty-free-image/155351346). RA Hand: Reproduced from BSIP / Universal Images Group via Getty Images (https://www.gettyimages.com/detail/news-photo/illustration-of-a-hand-suffering-from-rheumatoid-arthritis-news-photo/586117004?adppopup=true).

Rheumatoid arthritis is an autoimmune disorder characterized by inflammation of the synovial lining. Inflammation results in an increase in the number of synoviocytes and immune cells. As a result, the synovial membrane becomes hyperplastic, resulting in eventual cartilage and bone erosion (23). The pathogenesis of rheumatoid arthritis is illustrated in Figure 1.

Studies have suggested that RA risk is influenced by a genetic predisposition, environmental factors, or a combination of both. It is clear that immune cells, such as lymphocytes, neutrophils, and macrophages, play an important role in the pathophysiology of RA (24). Within the synovium of RA patients are macrophages and T cells that produce cytokines which promote inflammation and cell migration. Cytokines tumor necrosis factor-α (TNF-α), interleukin-1 (IL-1), and interleukin-6 (IL-6), produced by macrophages, and cytokine interleukin-17 (IL-17), produced by CD4+ T cells, are commonly involved in the inflammatory response and subsequent cartilage destruction.

These cytokines activate synoviocytes and cause them to proliferate, creating proteases in the synovial fluid, which lead to the breakdown of cartilage and hypertrophied synovial tissue, known as pannus (25). Pannus can be further exacerbated by angiogenesis. The additional blood supply to invaded cartilage and bone allows immune cells to infiltrate the joints, worsening the synovial hyperplasia (24). Cytokines also combine with receptor activator of nuclear factor kappa-β ligand (RANKL) to stimulate osteoclast activity, which leads to bone erosion. The expression of RANKL is also affected by T cells (26).

Synovial dendritic cells stimulate immune response by attracting T lymphocytes and activating antigen-specific T cells and, in turn, B cells. In this positive feedback loop, activated B cells stimulate CD4+ T cells, producing more cytokines (27, 28). B cell proliferation can also lead to the creation of plasma cells, which produce autoantibodies, including rheumatoid factor (RF) and anti-citrullinated protein antibodies (ACPAs) (29). These autoantibodies infiltrate the joint through newly developed blood vessels and are currently used in the diagnosis and prognosis of RA (30).

## Weight Control and RA

Studies show that excessive body weight increases the risk for developing RA (31). A 2011 report defined obesity as having higher than normal levels of all triglycerides found in adipose tissue, which can contribute to negative health outcomes such as increased inflammation, type 2 diabetes, insulin resistance, and cardiovascular disease (32). Excessive adipose tissue secretes pro-inflammatory cytokines (adipokines) into circulation, which can increase adipose tissue growth, leading to a positive feedback cycle of adipokine secretion and tissue inflammation (33).

Adipose tissue stores energy in the form of fat, which helps regulate several physiological processes such as insulin sensitivity, metabolism, and inflammation. However, having excess fat in adipose tissue and non-adipose tissue cells can hinder these physiological processes. Recent studies have found that increased fat inside cells is related to increased inflammation (32). In addition, the extra stress placed on weight-bearing joints by excess body weight further exacerbates inflammation in these patients; therefore, weight loss could be an effective therapy for individuals diagnosed with RA.

Multiple studies have concluded that RA patients who are overweight have worse outcomes than those with a normal body mass index (BMI≤24.9) (34, 35). The Canadian Early Arthritis Cohort (CATCH) study (*n* = 982) showed that being overweight or obese were independently associated with a decreased chance for achieving sustained RA remission. Overweight patients were 25 and 47% less likely to achieve sustained remission within 3 years, respectively (36). Similarly, the Nurses' Health Study I and II, two prospective cohort studies including a total of 239,131 U.S. female nurses, reported that being overweight at 18 years of age was associated with a 35% increased risk of developing RA and a 50% risk of developing seropositive RA in adulthood (33).

Weight loss could be useful for alleviating the effects of inflammation and obesity in RA patients. A 2018 retrospective analysis (*n* = 174) evaluated the association between weight loss and RA disease activity, and found that overweight individuals who lost ≥5 kg had a three-fold increased odds of disease activity improvement compared to those who lost <5 kg. The clinical disease activity scale quantifies RA disease activity from 0 to 76 (higher scores indicating higher disease activity) by measuring the number of tender and swollen joints as well as a physician and patient global assessment on a 0–10 scale. The study concluded that each kilogram of weight lost was associated with a clinical disease activity index improvement of 1.15 (*p* = 0.0026) (37). Sparks et al. investigated the effect of weight loss in RA patients after bariatric surgery (*n* = 53) and had similar findings. At a 12-month, post-surgery follow-up, 6% of patients had moderate/high disease activity compared to 57% at baseline. At the most recent follow-up (mean 5.8 years after surgery) 74% of patients were in remission compared to 26% at baseline. Furthermore, inflammatory markers were significantly lower at the 6-, 12-month, and most recent follow up visits of 5.8 ± 3.2 years following surgery (*p* < 0.05, *p* < 0.001, and *p* < 0.001, respectively) (38). Weight loss may be a key non-pharmacologic approach in reducing inflammation and RA disease activity.

In addition to the importance of weight management, a 2015 nested case-control study (*n* = 33,456) found an association between elevated serum cholesterol and the subsequent development of RA. Women diagnosed with RA at follow-up had higher total cholesterol levels at baseline compared with healthy controls (OR 1.42; 95% CI 1.08–1.87). However, this relationship was not observed in men (39). The positive association between total cholesterol levels and RA development in women suggests a possible mechanism related to female sex hormones. A low-fat, vegan diet, which reduces plasma cholesterol and has a hormone-stabilizing effect (40–42), may therefore help protect against the development of RA in women (39).

Vegetarian and vegan diets have been consistently shown to be effective weight loss and cholesterol-lowering strategies compared with other conventional calorie-restricted diets. Two meta-analyses of randomized clinical trials showed a benefit of vegetarian, especially vegan, diets on body weight, compared with other diets (43, 44). The strong evidence is supported by observational studies (9–11). Likewise, the evidence for the effectiveness of vegetarian, particularly vegan, diets in lowering total and LDL-cholesterol in clinical trials is consistent (45, 46) and is further supported by observational studies (47, 48). Elkan et al. (*n* = 66) observed reductions in BMI, LDL, and total cholesterol after both 3 and 12 months of a gluten-free vegan diet (*p* < 0.01). These results correspond with the improvements in Disease Activity Score of 28 joints, Health Assessment Questionnaires, and CRP levels (*p* ≤ 0.008) after 12 months (49). These findings suggest that by improving weight loss and lowering serum cholesterol levels, plant-based diets might improve RA symptoms and decrease the risk of developing the disease.

## Associations Between Diet and RA

### Diet and Inflammation

A 2015 study (*n* = 50) observed reductions in inflammatory scores in overweight or obese, otherwise healthy, participants randomized to a 2-month vegan, vegetarian, or pesco-vegetarian dietary intervention (*p* < 0.05) compared to those placed on a semi-vegetarian or omnivorous diet. Subjects in all five groups were counseled to choose low-fat foods, but only the vegan participants met the mean percentage energy from fat and saturated fat (≤30% energy from fat and ≤7% energy from saturated fat) recommendations. The researchers attributed this observation to the elimination of the leading sources of fat in the western diet (beef, cheese, milk, and poultry; 8). Diets high in fat and processed meat have been positive associated with inflammatory markers C-reactive protein (CRP), interleukin-6 (IL-6), and homocysteine, while diets high in whole grains and fruit have been inversely associated with these biomarkers (50). Likewise, vegetarian diets are negatively associated with CRP levels (*p* < 0.000) (51). Furthermore, a 3-week vegan lifestyle intervention resulted in a 33% reduction in CRP levels (*p* < 0.001), which was attributed to the anti-inflammatory components of the vegan diet, such as high fiber intake (>49 g/day) (12).

Research has found that a low-fat vegan diet improves RA symptoms, such as the degree of pain, joint tenderness, and joint swelling (13). A randomized clinical trial found that a gluten-free vegan diet decreases immunoglobulin G (IgG) in RA patients, an oftentimes elevated pro-inflammatory antibody (52). A Cretan Mediterranean Diet, rich in olive oil, cereals, vegetables, fruits, and legumes, also resulted in significant improvements in Disease Activity Score (DAS28), Health Assessment Questionnaire (HAQ), C-Reactive Protein (CRP), and swollen joint counts in patients with RA (53). However, further investigation is needed as previous research has also shown that a high-fat diet may change the composition of the gut bacteria and be linked to inflammation (54–56).

The naturally low-fat, fiber-rich components of a vegan diet might mediate the pathways that alleviate joint inflammation and pain, as observed through reduced CRP levels and improved inflammatory scores. These findings highlight the need for a randomized study that objectively measures biomarkers of inflammation related to plant-based dietary changes.

#### Relationship Between Inflammation and Protein Quantity and Source

Rheumatoid arthritis is a type of inflammatory polyarthritis, characterized by inflammation in more than four joints (57). Higher red meat intake has been positively associated with inflammatory polyarthritis (*p* = 0.04). Participants consuming the highest levels of red meat (OR 1.9, 95% CI 0.9–4.0), total meat (OR 2.3, 95% CI 1.1–4.9), and total protein (OR 2.9, 95% CI 1.1–7.5), displayed a higher risk for inflammatory polyarthritis when compared to participants with lower meat and protein intakes (58). These findings suggest that meat intake increases the risk of inflammatory arthritis.

Gögebakan et al. (*n* = 932) examined the effects of weight loss from varying dietary compositions on high sensitivity C-reactive protein (hsCRP) using data from the Diet, Obesity, and Genes study (DiOgenes) (59). After an initial weight loss of at least 8%, participants were randomized to 1 of 5 dietary interventions for 26 weeks. These interventions included: (1) low protein, low glycemic index; (2) low protein, high glycemic index; (3) high protein, low glycemic index; (4) high protein, high glycemic index; and (5) control diet based on national dietary guidelines. The initial calorie-restricted period resulted in a significant improvement of hsCRP, which is likely due to calorie restriction-stimulated activation of protective metabolic pathways, thus reducing inflammatory markers. Low-glycemic index diets resulted in a further decrease of hsCRP of −0.46 mg/L greater than in the high-glycemic-index groups. Similarly, hsCRP values decreased −0.25 mg/L more in the low-protein groups than in the high-protein groups. Gögebakan et al. postulate that the lower post-prandial glucose levels on low-glycemic index diets decrease inflammatory gene expression, resulting in reduced hsCRP levels (59).

A high-protein diet (28% protein, 43% carbohydrate, 13 g fiber) reduced insulin sensitivity by 12% while a high cereal fiber diet (17% protein, 52% carbohydrate, 43 g fiber) improved insulin sensitivity by 13% in 111 overweight and obese participants (60). Participants assigned to the high fiber diet displayed 25% higher insulin sensitivity than those on the high protein diet after the 6-week intervention (*p* = 0.008). These results indicate that high dietary protein (≥25–30% of energy) induces insulin resistance. Interestingly, insulin sensitivity was not significantly altered after 6 weeks of a mixed diet (23% protein, 44% carbohydrate, 26 g fiber). While dietary proteins are normally degraded by enzymes in the upper gut, these results indicate that cereal fibers may impede protein absorption in the small intestine. Thus, a low-glycemic, high-fiber and low-protein diet could mediate inflammation by decreasing pro-inflammatory gene expression and improving insulin sensitivity, even after significant reductions in inflammatory markers due to weight loss (59, 60). Ley et al. (*n* = 3,690) sought to examine the association between red meat intake and inflammatory biomarkers. Cross-sectional data from the Nurses' Health Study was analyzed and the association between total, unprocessed, and processed meat intake with CRP and adiponectin were measured. Greater total, unprocessed, and processed red meat intakes were associated with significantly higher plasma CRP concentrations and lower adiponectin levels for participants in the highest quintiles of these groups. Similarly, lower CRP values were associated with substituting a serving of total red meat with a combination of alternative protein sources (including poultry, fish, legumes, or nuts) (β = −0.106, *p* ≤ 0.02). However, these associations were no longer significant after adjustment for BMI, as well as medical and lifestyle variables (61).

BMI accounted for a statistically significant proportion of associations with these biomarkers. The results of this study indicate that while greater red meat intake is associated with higher plasma concentrations of inflammatory biomarkers in diabetes-free women, adiposity accounted for a statistically significant proportion of these associations. Excluding animal products has been shown to reduce adiposity and improve CRP and adiponectin levels (61).

These findings highlight some of the potential mechanisms by which vegan diets could improve inflammation in RA patients. Apart from eliminating leading triggers, reducing animal protein has been linked to lower inflammatory markers and increased insulin sensitivity. Research also suggests that the low-glycemic index and high fiber content of the diet could reduce inflammatory gene expression. Thus, a naturally anti-inflammatory vegan diet could improve RA symptoms.

#### Fat Intake and Inflammation

A 3-month Mediterranean dietary intervention which significantly increased the ratio of monounsaturated fats:saturated fats improved rheumatoid arthritis symptoms as measured by pain score and physical function (62). These findings suggest that saturated fat might be linked to poorer rheumatoid arthritis symptoms, while monounsaturated fats are associated with improved outcomes (63).

A 2013 study (*n* = 15) examined the acute antioxidant and inflammatory response following a high-fat meal in overweight participants over an 8 h period. Pro-inflammatory TNF-α and IL-6 concentrations increased significantly in response to the meal. TNF-α levels increased by 12 pg/mL (*p* < 0.001) in the first hour and remained significantly above fasting values throughout the 8 h. IL-6 levels constantly rose after the meal, doubling its basal value after 2 h (+0.3 pg/mL, *p* < 0.05) and reached a maximum concentration after 8 h. Likewise, total cholesterol levels increased throughout the post-meal study period and peaked at the 8 h point (+7 mg/dl; *p* < 0.01) (64).

High-fat meal ingestion also increased endogenous antioxidants, uric acid and thiols, indicating the presence of oxidative stress. The area under the curve of both uric acid and thiols was significantly correlated with the triglycerides area under the curve (Pearson coefficient 0.923). This concomitant antioxidant response to high-fat meal ingestion highlights the pronounced impact of dietary-induced inflammation, due to a single meal (64).

A moderate (3.68–13.67 g) or high (>13.67 g) reduction in saturated fatty acid consumption has been shown to reduce leptin, a pro-inflammatory adipokine, and increase adiponectin, an anti-inflammatory adipokine, in obese adolescents. Likewise a decrease in the pro-inflammatory leptin/adiponectin (L/A) and an increase in the anti-inflammatory adiponectin/leptin (A/L) ratio was observed after the reduction in saturated fatty acid consumption. A negative correlation between the change in SFA and adiponectin as well as A/L ratio (*p* < 0.05 for both) was observed. Participants with the greatest reduction of SFA increased their adiponectin levels by 50%, followed by 20% and 23% in the moderate and low SFA reduction groups, respectively (*p* ≤ 0.05) (65). This study demonstrates that a moderate change in SFA intake can yield significant changes in inflammatory measures.

The primary source of saturated fat in the U.S. is dairy products, followed by meat, eggs, and various processed foods (66). A low-fat, vegan diet is naturally free of animal products and low in SFA, potentially improving RA symptoms by up-regulating anti-inflammatory markers and down-regulating pro-inflammatory adipokines.

#### Complex Carbohydrates, Fiber Intake, and Inflammation

A 2009 systematic review (*n* = 554) sought to determine the influence of dietary fiber on CRP values in clinical trials (67). Increased fiber consumption, with corresponding altered fat intake and weight loss, was associated with lower CRP concentrations of 25–54% (*p* = 0.05) in six of the seven reviewed studies. The use of psyllium fiber supplementation in the seventh study did not result in lower CRP values, indicating that the effects of psyllium do not replicate those of a high-fiber diet (68). A significant decrease in inflammatory marker levels follows decreased fat intake (69), increased fiber consumption (70–72), and weight loss (69). Thus, a vegan diet, structured around low-glycemic foods and naturally high in fiber, has potential to lower inflammatory markers.

Dietary fiber is fermented by gut microbiota to produce short chain fatty acids (SCFAs), which have a beneficial effect on colonocytes (21, 73). Damage to colonocytes can result in intestinal permeability, endotoxemia, and inflammation. Thus, a diet rich in fiber provides an abundant energy source to colonocyte, reducing the risk of pathogens entering the bloodstream and inducing an inflammatory response.

These findings suggest that dietary fiber can reduce local and systemic inflammation, and that modulating effects on the gut bacteria composition, SCFA production, and intestinal barrier integrity could be involved.

### Microbiome and Inflammation

The gut may play a key role in the pathophysiology of RA. Permeability of the intestinal barrier allows for food components or bacterial endotoxins to enter the bloodstream. Absorption of endotoxins into circulation can trigger a systemic inflammatory response (74). This process may help explain the oftentimes elevated self-reactive antibodies and pro-inflammatory T lymphocytes in RA patients (13, 21, 75). The maintenance of the intestinal barrier is largely dependent on the composition of the gut microbiome. As discussed in the previous section, particular microbes ferment dietary fiber into the short chain fatty acids (SCFAs) that serve as the primary energy source for colonocytes. Certain SCFAs, particularly butyrate, have also been shown to ameliorate colonocyte DNA damage (21, 73). A microbiome lacking in diversity can result in lower concentrations of these SCFAs, and therefore, impair the intestinal barrier, allowing pathogens to enter the bloodstream, and inducing an inflammatory response (74). Previous research has shown that the microbiomes of RA patients not only lack microbial diversity but are dominated by *Prevotella copri* (75, 76). This bacterial species appears to lower the abundance of other beneficial species and thrive in untreated RA patients (75). Thus, the gut microbiome and dietary fiber intake might have a significant impact on RA disease activity.

The gut microbiome could mediate the connection between diet, inflammation, and RA, although these relationships remain speculative (77). Some studies suggest a connection between intestinal inflammation and joint inflammation (78). Kim et al. observed that a vegan diet lowers the relative abundance of *Enterobacteriaceae* in the gut, which in turn reduces fecal lipocalin-2 (Lcn-2), a sensitive biomarker of intestinal inflammation, within 28 days (54). Table 1 lists the type of correlation between bacterial species, dietary factors, and inflammation.

**Table 1:** Microbiota and inflammatory associations.

**Genus**	**Dietary association**	**Association with inflammation/RA**	**References**
*Ruminococcus*	**↑**Fruit and vegetables	**↓**Endotoxemia	(79)
	**↑**Non-digestible carbohydrates	**↓**Colorectal adenomas	(80)
*Roseburia*	↓High protein/low carbohydrate diet ↑Mediterranean diet ↓Animal protein	**↓**Inflammatory Bowel Disease **↓**Colorectal adenomas	(80)
*Bifidobacterium*	**↑**Non-digestible carbohydrates ↑Plant polyphenols ↑Low fat diet ↑Unsaturated fat ↑Date fruits ↑Mediterranean diet ↓Western diet ↓Beef	↓hs-CRP ↑Immune-modulation ↑Gut mucosal barrier	(80)
*Lactobacillus*	**↑**Non-digestible carbohydrates **↑**Plant polyphenols ↑Unsaturated fat ↑Mediterranean diet ↓High fat diet ↓Western diet	↑Anti-inflammatory activities ↓hs-CRP ↓Intestinal dysbiosis **↓**Inflammatory Bowel Disease	(80)

*↑Increase*.

*↓Decrease*.

### Elimination Diets in the Treatment of RA

As explored throughout this review, a growing body of research suggests that RA may have a gastrointestinal component and may even originate in the gut, at least for some individuals. In addition to other dietary considerations, antigenic load and sensitivities to specific foods may contribute to both the onset and severity of RA (81).

An early review by van de Laar et al. revealed that arthritic symptoms are associated with multiple gut-related conditions, including celiac disease, intestinal bypass, and inflammatory bowel disease. Moreover, mast cells, which are activated in response to foreign antigens, often in a process mediated by immunoglobulin E (IgE), are present in elevated numbers in the synovial tissues of patients with RA (82, 83). Even more telling, cross-reactive antibodies to various foods are found in the small intestine of those with RA at markedly higher levels than in healthy individuals (84).

Multiple studies have found improvements in RA patients placed on an elemental diet in which all antigenic proteins are eliminated from the patient's diet. The individual is given, as a sole source of nutrition, a complete formula in which all proteins have been broken down into free amino acids. A study conducted by Podas et al. found that placing patients with RA on an elemental diet was as effective as 15 mg/day of oral prednisolone over a 2-week period (81). However, symptoms returned upon cessation of the elemental diet, just as for cessation of prednisolone. Similar benefits were not seen for patients on a peptide diet containing protein fragments 3–6 amino acids in length (85).

Elimination diets, which remove one or more foods likely to trigger symptoms, have also been shown to induce clinical improvement in RA patients in clinical trials (52, 86). These improvements disappear when patients resume their normal diet (86).

However, response to elimination diets is highly individualized. Darlington et al. reported that some RA patients were “good responders.” These individuals, who reported feeling “better” or “much better” after an elimination diet (75% of respondents), showed profound improvements in all or nearly all measures of disease activity. Interestingly, after analyzing a number of personal attributes, the only factor significantly associated with a good response to the elimination diet was a family history of atopy (86).

One challenge in studying elimination diets for RA is that, as for food allergies, trigger foods are often unique to each individual (87). Multiple methods have been tried to identify individual food sensitivities, with varying degrees of success. Skin prick testing (SPT) is a tool used to identify IgE antibody response to a stimulus, but does not consistently correspond with reactions to offensive foods (88).

However, SPT and oral food challenges have both identified foods capable of worsening RA symptoms in some individuals. For example, SPT was used to identify corn, wheat, coffee, soybeans, and other foods as possible triggers in 20 RA patients who demonstrated reactivity in SPT (Note: the researchers did not test dairy products or red meat due to bovine spongiform encephalopathy concerns). Of the 18 patients who subsequently underwent an elimination period wherein they omitted common food allergens, followed by a challenge with foods they reacted to in SPT, 13 (72%) experienced worsening symptoms after reintroduction of SPT-positive foods (89).

Darlington et al. used elimination and oral food challenge to identify foods capable of inducing symptoms in RA patients. Forty-eight patients undertook a 6-week elimination diet; forty-one were found to have foods that triggered symptoms. Foods triggering symptoms for reactive patients are described in Table 2 (90).

**Table 2:** Foods inducing symptoms in food-reactive RA patients (*n* = 41).

**Food**	**Percent of patients affected**	**Food**	**Percent of patients affected**
Corn	57	Malt	27
Wheat	54	Cheese	24
Bacon/pork	39	Grapefruit	24
Oranges	39	Tomato	22
Milk	37	Peanuts	20
Oats	37	Sugar (cane)	20
Rye	34	Butter	17
Eggs	32	Lamb	17
Beef	32	Lemons	17
Coffee	32	Soy	17

*From Darlington and Ramsey (90)*.

Serum levels of food-specific antibodies and even rectal food protein challenge have also been tried; however, these methods have largely failed to identify a reliable link between specific foods and clinical symptoms (87). Additional foods have been implicated in some individuals using these and other methodologies. For example, the level of antibodies specific to *Saccharomyces cerevisiae* (baker's or brewer's yeast) in the blood of RA patients strongly correlates with C-reactive protein levels and erythrocyte sedimentation rate, both markers of inflammation (91).

Animal foods, including milk, eggs, and dairy, have also been found to be particularly problematic for RA patients, as evidenced by studies showing symptomatic improvement with a vegan diet (13, 52). Certain cereals may also pose problems in addition to animal products. Confirming findings from food challenge studies, one trial found strikingly higher levels of antibodies to milk, egg, pork, and codfish antigens, along with wheat, oat, and soy antigens, in the jejunal fluid of RA patients when compared to controls. Given evidence suggesting these results were not due to increased intestinal permeability from NSAID exposure, study authors concluded that mucosal immune activation in the intestine could play a role in the pathogenesis of RA (84).

However, a 2009 Cochrane review found “uncertain” effects of elimination and vegan diets as a result of inadequate data reporting (92); little if any research has since been published on the effects of elimination diets on RA. The lack of recent research on this topic is concerning, since emerging research has shown that diets eliminating specific foods can be effective for other inflammatory and autoimmune conditions, such as Crohn's disease (93, 94) and eosinophilic esophagitis (95–98). Eliminating gluten has also been found to not only ameliorate intestinal signs and symptoms in celiac disease (CD) but also to improve arthritis/arthralgia in some CD patients (99, 100).

The relationship between diet and RA is complex, and foods that trigger reactions in patients are individualized and therefore can be challenging to detect. However, a vegan diet comprised of fruits, grains, legumes, and vegetables can be a beneficial start for RA patients. In addition to being associated with lower BMI and greater fiber intake, this diet may help improve symptoms by eliminating many common trigger foods. Further elimination may be beneficial depending on the individual.

## Conclusion

Several studies have shown that joint pain and other RA symptoms may be modified by dietary factors. Excessive body weight and diets that include animal products (e.g., dairy, red meat) exacerbate the RA symptoms likely due to their pro-inflammatory effects. In contrast, diets rich in vegetables, fruits, and fiber are associated with lower BMI, have anti-inflammatory properties and help reduce pain and inflammation in these patients. Studies have shown that dietary fiber found in these plant-based foods can improve the gut bacteria composition and increase the bacterial diversity in RA patients, potentially reducing inflammation and joint pain. Moreover, although some of the trigger foods in RA patients are individualized, a vegan diet comprised of fruits, whole grains, legumes, and vegetables, can improve symptoms by eliminating many common trigger foods. Elimination of additional food triggers may be necessary depending on the individual food sensitivities. Further research is needed to test the effectiveness of plant-based diets on joint pain, inflammation, and quality of life in patients with RA.

## Author Contributions

JA and HK contributed organization of the manuscript. MC drafted the manuscript and composed the outline. JA, HK, ER, WY, SD, MC, NBu, and LC wrote sections of the manuscript. NBa reviewed and approved the submitted version. All authors had full access to data and revised and approved the manuscript for publication.

### Conflict of Interest Statement

The authors declare that the research was conducted in the absence of any commercial or financial relationships that could be construed as a potential conflict of interest.
